# Conventional and Digital Impressions for Fabrication of Complete Implant-Supported Bars: A Comparative In Vitro Study

**DOI:** 10.3390/ma16114176

**Published:** 2023-06-04

**Authors:** Samanta N. V. Vieira, Matheus F. Lourenço, Rodrigo C. Pereira, Esdras C. França, Ênio L. Vilaça, Rodrigo R. Silveira, Guilherme C. Silva

**Affiliations:** 1 Department of Restorative Dentistry, School of Dentistry, Universidade Federal de Minas Gerais, Belo Horizonte 31270-901, MG, Brazil; samanta.vinagre@gmail.com (S.N.V.V.); esdrasodonto@gmail.com (E.C.F.); elvilaca@gmail.com (Ê.L.V.); rodrigorsilveira@hotmail.com (R.R.S.); 2 Private Practice, Belo Horizonte 30130-007, MG, Brazil; matfralou@gmail.com; 3 Universo, Belo Horizonte 31140-320, MG, Brazil; rodrigocaillaux1976@gmail.com

**Keywords:** CAD-CAM, implant-supported prostheses, dental implants, zirconia, bone implant

## Abstract

Obtaining accurate models and well-fitting prostheses during the fabrication of complete implant-supported prostheses has been a significant challenge. Conventional impression methods involve multiple clinical and laboratory steps that can lead to distortions, potentially resulting in inaccurate prostheses. In contrast, digital impressions may eliminate some of these steps, leading to better-fitting prostheses. Therefore, it is important to compare conventional and digital impressions for producing implant-supported prostheses. This study aimed to compare the quality of digital intraoral and conventional impressions by measuring the vertical misfit of implant-supported complete bars obtained using both types of techniques. Five digital impressions using an intraoral scanner and five impressions using elastomer were made in a four-implant master model. The plaster models produced with conventional impressions were scanned in a laboratory scanner to obtain virtual models. Screw-retained bars (n = five) were designed on the models and milled in zirconia. The bars fabricated using digital (DI) and conventional (CI) impressions were screwed to the master model, initially with one screw (DI1 and CI1) and later with four screws (DI4 and CI4), and were analyzed under a SEM to measure the misfit. ANOVA was used to compare the results (*p* < 0.05). There were no statistically significant differences in the misfit between the bars fabricated using digital and conventional impressions when screwed with one (DI1 = 94.45 µm vs. CI1 = 101.90 µm: F = 0.096; *p* = 0.761) or four screws (DI4 = 59.43 µm vs. CI4 = 75.62 µm: F = 2.655; *p* = 0.139). Further, there were no differences when the bars were compared within the same group screwed with one or four screws (DI1 = 94.45 µm vs. DI4 = 59.43 µm: F = 2.926; *p* = 0.123; CI1 = 101.90 µm vs. CI4 = 75.62 µm: F = 0.013; *p* = 0.907). It was concluded that both impression techniques produced bars with a satisfactory fit, regardless of whether they were screwed with one or four screws.

## 1. Introduction

The functional and esthetic rehabilitation of fully edentulous patients using implant-supported prostheses has proved to be predictable in the long term [1]. However, biological and mechanical failures of implants and prostheses can still occur [2,3,4]. Complications related to implant-supported prostheses may include misfit or lack of passivity to the implant or prosthetic abutment [5,6]. It is considered unfeasible to achieve a passive fit of the prostheses due to inherent distortions in various clinical and laboratory procedures [5,6,7,8,9]. Nevertheless, it is important to aim for excellent accuracy in implant rehabilitations because inadequate adaptation in implant-supported prostheses can result in increased stress and mechanical problems, such as loosening or fracture of retention screws [5,6,9]. A number of clinical and laboratory variables can contribute to the production of poorly fitting prostheses, with the transfer of implant position to the working model being a critical factor [8,9,10].

Several traditional impression techniques utilizing different impression materials, transfer copings, and trays have been assessed for their ability to produce models capable of fabricating prostheses with a high level of fit [8,10]. Among conventional techniques, elastomeric impressions using an open tray with splinted transfers appear to result in prostheses with superior fit, making it the preferred conventional technique [10]. However, conventional techniques, besides being time-consuming, usually result in models with some distortion and prostheses with some degree of misfit [7,8,9,10]. Although it is not possible to fabricate implant-supported prostheses without some degree of mismatch, current conventional techniques allow their fabrication to the extent that they can be long-lasting [2,3,4]. Moreover, through the combination of digital CAD-CAM techniques, conventional impression methods can generate satisfactory prostheses, even in full-arch complex cases [11].

The use of intraoral digital impressions could be an approach to eliminate or minimize clinical and laboratory steps that usually introduce errors in the fabrication of implant-supported prostheses [8,9]. Digital impression involves acquiring 3D images of the implant/abutment and surrounding tissues using an intraoral scanner. A scan body with easily readable geometry is screwed onto the implant/abutment to capture its image. The scan body identifies the type of connection and the depth of the implant/abutment [8]. This technique has been used frequently with promising clinical results [10,12], especially for tooth-supported, implant-supported single crowns [13], and to generate milled structures [14]. Laboratory studies and reviews comparing digital and conventional impressions for single implants have shown similar outcomes [15,16,17,18]. Furthermore, digital impressions can be more time- and cost-effective [19,20,21], more acceptable by patients [22], and well-accepted by students and practitioners [23], justifying the growing use of this digital technology in dentistry.

However, in multiple implants [24], especially for full-arch cases [17,25,26,27,28,29,30,31,32,33,34,35,36,37,38], intraoral digital impressions still lack predictability. Numerous methods have been used to assess the quality or accuracy of intraoral digital impressions for multiple implants. The most common is the use of coordinate-measurement machines and the analysis of 3D coordinate axes obtained through superimposition of STL files generated using a laboratory scanner (control) and intraoral scanner and elastomeric impression tests [12,14,16,24,25,26,29,30,31,32,33]. They can measure the 3D deviation and, consequently, the trueness and precision between the experimental models and the master model without the need to construct prostheses. Other studies analyzed the quality of fit directly in the prostheses utilizing radiographic analysis [36,38], strain recording [15], optical microscopy [34], and the Sheffield test [36,38]. Based on the findings of some of these studies evaluating digital impression, it has been suggested that it may not be suitable for routine clinical use in full-arch prostheses [16]. Additionally, some specific intraoral scanner models were not recommended for use in full arches [32]. Moreover, caution is advised when using digital impressions for multiple implants with an angulation exceeding 15°, as the results may be unreliable [33]. Even in more recent studies, authors have demonstrated that intraoral scanning can exhibit errors that may impact clinical success [31,33]. Despite these limitations, other studies have presented promising outcomes regarding intraoral scanning for implant full-arch rehabilitations. An in vitro study has demonstrated smaller marginal discrepancy under optical microscopy of complete implant-supported structures obtained with digital impressions [34]. A recent laboratory study has found significantly less 3D deviation on models in digital groups compared to a conventional polyether splint open-tray impression [30]. A clinical retrospective study showed that implant deviations found between both the full-arch digital and conventional impressions lie within the clinically acceptable threshold [35]. Systematic reviews showed acceptable [36] or even higher accuracy for intraoral scanning in implant full-arch models [37]. More recently, a clinical study which compared full-arch implant-supported prostheses made using conventional and digital intraoral impressions found no differences in the fit quality between both methods. Nevertheless, digital analysis resulted in prostheses with better accuracies [38]. Thus, despite the evident progress observed in recent studies, there is still no consensus on whether intraoral digital impressions can be regularly used to produce complete implant-supported dentures.

While the digital impression technique for teeth and single implant-supported prostheses is well-established, there is still a need for further development and discussion regarding intraoral digital impressions in full arches [13,17,18,28,29,30,31,33,36,37]. Therefore, this study aimed to compare, in vitro, digital impressions using an intraoral scanner and conventional impressions using elastomers by measuring the vertical misfit in the abutment/prosthesis interface in full-arch implant-supported bars obtained via both methods. The null hypothesis is that there are no differences in misfit in the structures designed on models obtained using digital and conventional impressions.

## 2. Materials and Methods

**Figure 1:**
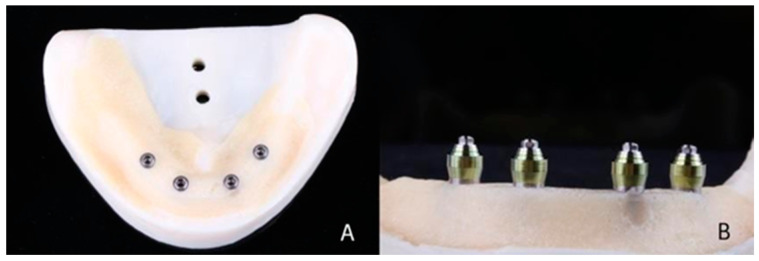
(**A**) Master model. Implants numbered 42, 41, 31, and 32, from left to right in image. (**B**) Multi-unit-type abutments screwed onto implants.

**Figure 2:**
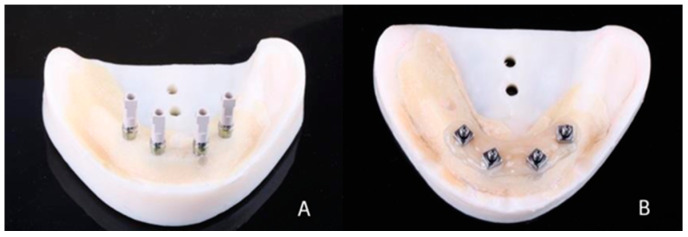
(**A**) Scan bodies screwed onto abutments. (**B**) Open-tray transfers screwed onto implants and splinted with bis-acrylic resin.

**Figure 3:**
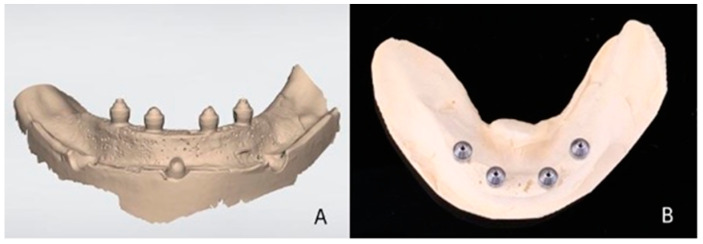
(**A**) Digital model generated using intraoral scanner. (**B**) Plaster model obtained in conventional impression group.

A master model was made by installing 4 titanium external hexagon implants (SIN Implant System, São Paulo, Brazil) (diameter: 4.1 mm; length: 10 mm) in the interforaminal region of a training model (Implant Model, Pronew, São Gonçalo, Brazil) using a milling machine (F1, Degussa, Dusseldorf, Germany) to ensure inter-implant distance (5 mm) and parallelism between them (Figure 1A). Over the implants, straight multi-unit abutments (SIN Implant System) (height: 3 mm) were screwed with a torque of 32 Ncm (Figure 1B). The abutments were numbered 42, 41, 31, and 32 (FDI). Scan bodies for multi-unit abutments (SIN Implant System) were screwed (10 Ncm) onto the abutments (Figure 2A), and the model was scanned using an intraoral scanner (Trios 3, 3Shape, Copenhagen, Denmark) according to the scanning technique recommended by the manufacturer. Five virtual models were made with five digital impressions (Figure 3A).

Five impressions of the master model were made using customized open trays (Vivid ExelTray LC, Pearson Dental, Sylmar, CA, USA) to create five conventional plaster models. Open-tray transfers (SIN Implant System) were screwed (10 Ncm) onto each abutment and splinted using self-curing bisacrylic resin (Luxatemp Star, DMG, Hamburg, Germany). After 10 min, the resin was sectioned with a carborundum disk and rejoined with a small portion of the bisacrylic resin (Figure 2B). Poly-vinyl siloxane (VPS) adhesive (Universal Tray Adhesive, Zhermack, Badia Polesine, Italy) was applied to the inner part of the tray, and after 8 min of drying, the tray was loaded with monophase VPS dispensed with automix cartridges (Honigum Mono, DMG, Hamburg, Germany) (Pentamix Lite, 3M Espe, Neuss, Germany) and placed on the master model. After 7 min, the transfers were unscrewed, and the mold was removed from the master model and stored for 30 min, protected from direct light at room temperature and humidity. Subsequently, multi-unit abutment analogs (SIN Implant System) were screwed to the transfers, and 80 g of type IV plaster (Fuji Rock EP, GC Europe, Leuven, Belgium) was vacuum spatulated according to the manufacturer’s specifications and poured over the mold using a gypsum vibrator. After 1 h, the transfers were unscrewed, and the model was separated from the mold (Figure 3B). The models were stored away from direct light at room temperature and humidity. Scan bodies (SIN Implant System) were screwed (10 Ncm) onto the abutments of the 5 plaster models and scanned using a laboratory scanner with an accuracy of 6 µm (Map 200, AmannGirrbach, Klobach, Austria), generating 5 virtual models.

**Figure 4:**
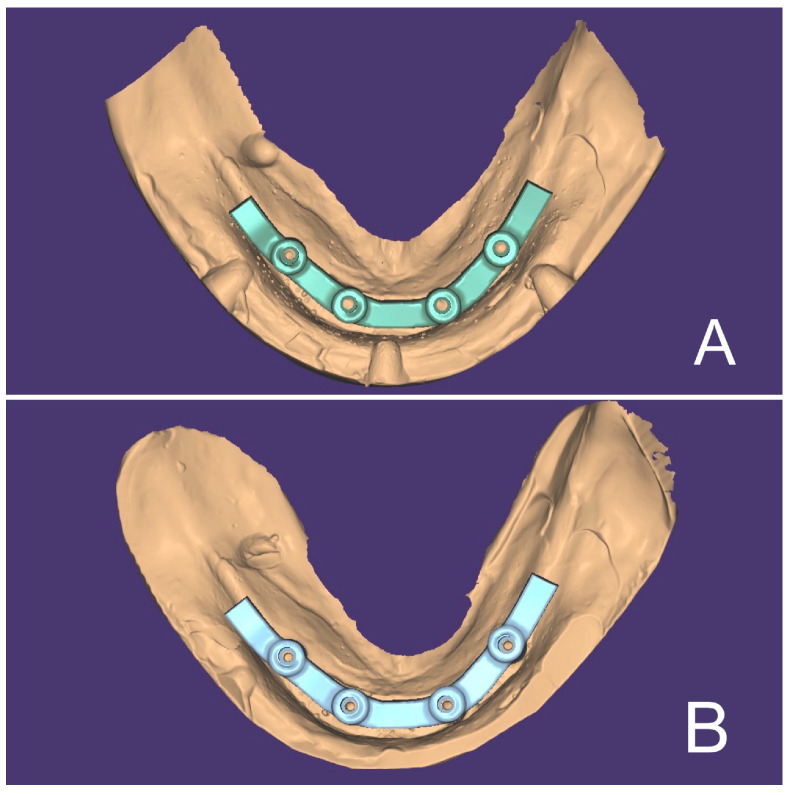
(**A**) Digital bar designed on master model: digital impression group. (**B**) Digital bar designed on master model: conventional impression group.

Each of the 5 virtual models obtained by digital impressions was transferred to the CAD software (DentalCad 2016, Exocad, Darmstadt, Germany). On each model, a bar-type screw-retained complete prosthetic framework was designed (Figure 4A). The external anatomy of the framework was saved and used for each design on each subsequent model. The 5 virtual models generated via the plaster models were worked in the same way as the models produced via the digital impressions with the screw-retained bars design (Figure 4B).

**Figure 5:**
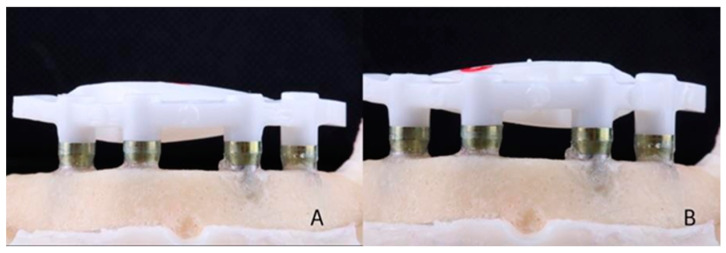
(**A**) Zirconia bar screwed on master model: digital impression group. (**B**) Zirconia bar screwed on master model: conventional impression group.

Each file was sent to a 5-axis milling machine (Motion 2, AmannGirrbach, Klobach, Austria), where the zirconia framework (Ceramill Zi, AmannGirrbach, Klobach, Austria) was milled. For each bar, a new set of burs was used. After milling, the bars were sintered (Ceramill Therm, AmannGirrbach, Klobach, Austria) according to the manufacturer’s recommendations (Figure 5A,B). Finally, 5 bars from the digital group (DI) and 5 bars from the conventional group (CI) were obtained (n = 5).

Each zirconia bar was positioned on the 4 abutments of the master model. For the first analysis under a microscope, only 1 screw on implant number 31 was torqued (15 Ncm). For the second measurement, all 4 screws were torqued (15 Ncm) onto each abutment. The master model with each bar was taken to the scanning electron microscope (SEM) (6360LV, JEOL JSM, Tokyo, Japan), always in the same position. The vertical misfit, which represents the difference in alignment along the vertical plane (*Z*-axis) and is consistently observed as a gap [9,39], was assessed at 1000× magnification in a frontal view for all 4 abutments of each bar, using either 1 or 4 screws.

**Figure 6:**
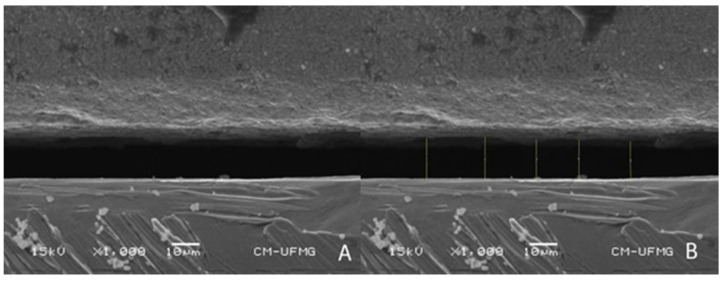
(**A**) Abutment–bar interface SEM image of abutment 42, digital bar 2 (DB2). (**B**) Abutment–bar interface SEM image of abutment 42, digital bar 2 (DB2), with 5 vertical lines used to measure the mean misfit of this abutment. 1000× magnification.

Public domain image processing and analysis software (ImageJ 1.52, National Institutes of Health, Bethesda, MD, USA) was used for the measurements. From the SEM-generated images at 1000× magnification, 5 equidistant vertical measurements were taken on the edge of the buccal surface of the mismatch (gap) between each abutment and bar (Figure 6A,B). The mean of these 5 measurements was considered the final vertical misfit for each abutment (32, 31, 41, and 42). The mean of these misfit values of the 4 abutments resulted in the final mean misfit of that bar. The mean of the misfit values of the 5 bars resulted in the final mean value of a group. As each bar was submitted to two different measurements (with 1 and 4 screws), the digital group (DI) was subdivided into DI1 and DI4, and the conventional group (CI) between CI1 and CI4, each containing the 5 respective bars of that group, measured with 1 and 4 screws, respectively.

The sample size was determined using the Kruskal–Wallis test, with a confidence level of 95% and a 5% margin of error, based on the “n” value from a similar study [14]. The calculation was performed using the Epi Info statistical package (CDC, Atlanta, GA, USA) and resulted in a minimum requirement of n = 5 in each group to meet the assumptions of the non-parametric Kruskal–Wallis test.

The means obtained in each group were subjected to the Kolmogorov–Smirnov test, which confirmed that they did not significantly differ from those that are normally distributed, indicating a normal distribution (*p* = 0.5427). Levene’s test showed a homogeneity of variance (*p* = 0.1859). Additionally confirmed were the assumptions of the homogeneity of variance and normal distribution of the sample. The values obtained for vertical misfit for each group of bars were statistically compared with the Analysis of Variance (ANOVA) test (SPSS, IBM, Armonk, NY, USA), considering a 95% confidence interval (*p* < 0.05).

## 3. Results

For the digital group screwed with one screw (DI1), a vertical misfit of 94.45 μm (±53.94) was found; for the digital group with four screws (DI4), a vertical misfit of 59.43 μm (±27.22) was found. For the conventional group screwed with one screw (CI1), a vertical misfit of 101.90 μm (±63.66) was found; for the conventional group with four screws (CI4), a vertical misfit of 75.62 μm (±71.59) was found. Misfit values, means, and standard deviations for each bar for DI1, DI4, CI1, and CI4 are described in Table 1.

There were no statistical differences between the misfit values found in bars generated using digital and conventional impressions and screwed with one or four screws. Additionally, there were no statistical differences when comparing the bars screwed with 1 or 4 screws within the same group (Table 2).

## 4. Discussion

The null hypothesis was accepted, as there were no statistical differences in the vertical mismatch of the bars generated using digital and conventional methods.

The quality of fit of implant-supported prostheses is one of the fundamental requisites for long-term mechanical success [5,6]. However, it is challenging to obtain passive proper fit [7,8,9,14,34], particularly in monolithic structures such as zirconia that do not allow for sectioning and welding. Conventional techniques for obtaining working models involve clinical–laboratory steps that can introduce small errors into the process, such as polymerization and elastic recovery of elastomers, movement of transfer copings, and plaster expansion [10,18,34]. In contrast, intraoral digital impressions have the theoretical potential to minimize these errors [8,9,36]. It seems that the open tray with splinted transfers associated with high-precision elastomers such as VPS or polyether is the most accurate technique to obtain working models for implant-supported prostheses [10], and the present study used this method for the conventional impression group. Studies and reviews have attempted to elucidate the feasibility of using intraoral digital impressions in full-arch implant-supported prostheses cases, with general agreement on the challenges associated with the technique [12,13,14,17,24,25,26,27,28,29,30,31,32,36,37,38]. The large area to be scanned increases the risk of minor deviations accumulating during the formation of three-dimensional (3D) images [29]. Additionally, superimposition of images often occurs in the absence of fixed anatomic references and the presence of similarities in the morphology of scan-bodies [29,34]. Mandible/tongue movements, excessive salivation, and operator experience are other complicating factors [33,38]. In the present in vitro study, the scanning process was performed in a parallel-implants stable model without movements, tongue superimposing, moisture, or saliva, leading to caution in interpreting results. Another limitation of the present study is that the results obtained from the digital group refer to a specific intraoral scanner (Trios 3, 3 Shape), and there is significant variation in accuracy and predictable errors between different intraoral scanners [30,31,32].

The present study compared the two impression methods using the SEM to measure the vertical mismatch [39] in bars produced using these techniques. Dimensional analysis based on direct mismatch measurements of a vertical gap is a valid technique for evaluating the quality of methods for fabricating implant-supported prostheses [6,9]. Optical microscopy could potentially provide adequate misfit measurements at 1000× magnification, promoting resource efficiency. However, the presence of birefringence caused by the proximity of dissimilar materials with distinct optical properties (zirconia bar and titanium abutments) often led to blurred images, making it difficult to accurately draw measurement lines. Therefore, scanning electron microscopy (SEM) was used as it offered clear and high-resolution images, enabling precise delineation of measurement lines. The obtaining of misfit vertical values in μm ensures a more clinical-oriented understanding and discussion because it is possible to observe the actual mismatch values of the prosthetic structure. Consequently, it allows for comparisons with levels of misfit found in the literature that are considered adequate. There is no consensus on the maximum value considered adequate considering the misfit. Several studies and reviews considered that misfit values up to 150 μm are clinically acceptable [9,14,18,34,36], while others suggested that this limit would be 200 μm [12]. The consensus is that the misfit should be minimized to the lowest extent possible, thereby reducing the risks associated with mechanical and biological complications. In this study, to minimize the usual inaccuracies in the margins of the zirconia bars [9] and eliminate the potential influence of milling bur efficiency in the resulting misfit, a new set of burs was used for milling each bar. The results obtained using both impression methods in this study showed a level of adaptation within the limits considered adequate by the literature: DI1 = 94.45 ± 53.94 μm; DI4 = 59.43 ± 27.22 μm; CI1 = 101.90 ±63.66 μm; CI4 = 75.62 ± 71.59 μm. Another laboratory study which compared full-arch prostheses found a mean marginal discrepancy under optical microscopy readings of 135.1 µm for the conventional group and 63.14 µm for the digital technique [34]. Unfortunately, direct comparison with the values in the results of other studies that compared conventional and digital impressions in implant-supported full-arch prostheses was not possible, as they measured 3D deviation [12,14,16,24,25,26,29,30,31,32,33] rather than the gap between the abutment and the prosthesis. Being an in vitro study, the misfit values obtained in the present study cannot be directly extrapolated to the clinical reality. However, it can be inferred that both impression techniques resulted in satisfactory prostheses.

Comparisons between DI1 × CI1 (*p* = 0.840) and DI4 × CI4 groups (*p* = 0.651) showed no statistically significant differences. Thus, digital impressions were as effective as conventional impressions in terms of the quality of the vertical adaptation of the prosthetic structure. This finding is consistent with the results of other studies comparing the two methods by using prostheses [12,15,16,38]. Moreover, systematic reviews concluded that digital full-arch implant impressions may be as accurate as conventional impressions [36] or better [37], whereas some laboratory studies reported superior results for digital impressions [30,34]. A recent clinical study also found that both techniques would be in an acceptable range of precision for full-arch implant-supported prostheses [35]. Contrarily, a laboratory study suggested that the digital method was less accurate [24], and a systematic review could not provide clinical guidelines on the most accurate impression technique due to limited high-quality evidence studies [18]. It is important to analyze which scanners were used in the studies that reported lower accuracy for the digital impression [16,18,24], as more recent scanners tend to provide better results [30,32,38]. Additionally, the scan body type and scanner model generate different results [30,36]. Thus, there is no clear conclusion on the superiority of one technique over the other, given that numerous variables make different results equally applicable [33]. However, there is a clear trend in more recent studies to generate better results, possibly due to the utilization of more modern scanners.

Images were acquired with bar adaptation using one or four screws to minimize the possibility of improved fit due to tensioned approximation by tightening the screws [7]. Additionally, zirconia was used as bar material because of its higher stiffness compared to metallic alloys, which may prevent a forced adaptation when tightening the screws [9]. The intragroup comparison between DI1 × DI4 (*p* = 0.123) and CI1 × CI4 groups (*p* = 0.907) showed no statistically significant differences. This indicates that the adaptation of the bars to the abutments occurred passively in most parts, given the lack of significant interference of the number of torqued screws (one or four) in the perceived adaptation levels. This result is consistent with the aim of this study, as there was no significant interference of tensile stress and consequent micro strains generated by the forced adaptation using the screws. It is important to emphasize that all bars were screwed onto the same master model. Thus, as in any study employing a master model to assess the adaptation of prosthetic structures, micro deformations may occur in the master model, potentially influencing the measurement outcomes. To minimize the risk of deformation, care was taken to screw each bar onto the master model only once, using the torque recommended by the manufacturer.

Although there were no significant differences in fit, digital impressions were considered by other studies to be more effective in terms of laboratory time [19,20,21], cost [20], and patient comfort [22,27]. Thus, it is expected in the short-term to see the improvement of hardware and software systems for obtaining better digital impressions in full-arch implant cases. Further comparative clinical studies are needed that encompass the numerous variables in both impression techniques to achieve conclusive results.

## 5. Conclusions

Within the limitations of an in vitro study, the present results indicate that intraoral digital impressions employed in the fabrication of mandibular full-arch prosthetic bars attained a satisfactory degree of vertical adaptation, comparable to bars manufactured using conventional impressions. These findings suggest the potential of intraoral digital impression techniques as viable alternatives to conventional approaches for prosthetic structure production. Nonetheless, additional clinical research and long-term assessments are imperative to validate these findings and appraise the comprehensive clinical performance of intraoral digital impressions in full-arch implant-supported protheses.

## Figures and Tables

**Table 1:** Mean value of vertical mismatches found in each group, in μm, with respective standard deviation (SD) value.

Bar	Digital Impression (DI)		Conventional Impression (CI)	
	1 screw (DI1)	4 screws (DI4)	1 screw (CI1)	4 screws (CI4)
1	103.80	38.47	167.39	31.36
2	39.04	34.54	60.18	34.95
3	64.38	55.42	20.27	14.02
4	84.16	66.37	100.48	114.57
5	180.88	102.34	161.21	183.22
	**94.45 (mean)**	**59.43 (mean)**	**101.90 (mean)**	**75.62 (mean)**
	**53.94 (SD)**	**27.22 (SD)**	**63.66 (SD)**	**71.59 (SD)**

SD, standard deviation; DI1, digital group with 1 screw; DI4, digital group with 4 screws; CI1, conventional group with 1 screw; C14, conventional group with 4 screws.

**Table 2:** F value and *p* value after Analysis of Variance (ANOVA).

Groups	F Value	*p* Value
DI1 vs. DI4	2.926	0.123
CI1 vs. CI4	0.013	0.907
DI4 vs. CI4	2.655	0.139
DI1 vs. CI1	0.096	0.761

DI1: digital group with 1 screw; DI4: digital group with 4 screws; CI1: conventional group with 1 screw; C14: conventional group with 4 screws.

## Data Availability

The data presented in this study are available on request from the corresponding author.
